# Evolutionary rescue on genotypic fitness landscapes

**DOI:** 10.1098/rsif.2023.0424

**Published:** 2023-11-15

**Authors:** L. M. Wahl, Paulo R. A. Campos

**Affiliations:** ^1^ Department of Mathematics, Western University, London, Ontario, Canada N6A 5B7; ^2^ Departamento de Física, Centro de Ciências Exatas e da Natureza, Universidade Federal de Pernambuco, Recife-PE 50670-901, Brazil

**Keywords:** evolutionary rescue, Fisher's geometric model, moving optimum, stochastic process

## Abstract

Populations facing adverse environments, novel pathogens or invasive competitors may be destined to extinction if they are unable to adapt rapidly. Quantitative predictions of the probability of survival through adaptation, evolutionary rescue, have been previously developed for one of the most natural and well-studied mappings from an organism’s traits to its fitness, Fisher’s geometric model (FGM). While FGM assumes that all possible trait values are accessible via mutation, in many applications only a finite set of rescue mutations will be available, such as mutations conferring resistance to a parasite, predator or toxin. We predict the probability of evolutionary rescue, via de novo mutation, when this underlying genetic structure is included. We find that rescue probability is always reduced when its genetic basis is taken into account. Unlike other known features of the genotypic FGM, however, the probability of rescue increases monotonically with the number of available mutations and approaches the behaviour of the classical FGM as the number of available mutations approaches infinity.

## 1. Introduction

In evolutionary rescue, a population facing extinction due to an adverse environmental change survives through rapid evolution [1–3]. Typically, an evolutionary rescue scenario involves a population that faces an abrupt environmental change, such that the population size is suddenly in decline. The population can then be ‘rescued’ by having, or creating, a rare genotype that is better adapted to the new environment. Evolutionary rescue is thus an essential concept not only in evolutionary theory [4], but also in conservation biology, since species threatened with extinction due to habitat fragmentation and destruction, as well as climate change, may survive through adaptation [5]. Understanding the mechanisms of evolutionary rescue requires the integration of both ecological and evolutionary responses of populations to a changing environment [1,6].

The fate of populations under stressful environmental conditions is determined by a complex interplay among demographic, genetic and extrinsic factors. The size of the initial population is a crucial determinant of evolutionary rescue [7–9], along with the rate of population decline, which is determined by the degree of maladaptation or level of stress in the adversely changed (or changing) environment [10–12]. The population is likely to face a geometrical decline in abundance if sufficiently maladapted [13], and may decline to critically small sizes at which it will be highly susceptible to extinction via demographic stochasticity [1]. On the other hand, the decay of the wild-type population also releases competition between existing mutants to facilitate rescue [14,15].

The mutation supply of beneficial mutations is another crucial factor that is impacted by both population size and mutation rate [8]. Standing genetic variation (genotypes that exist before the environmental change) and the rate of de novo mutation from the declining wild-type population are the main genetic factors that affect the probability of rescue [13,16]. In addition, a positive effect of local dispersal has been demonstrated in structured populations, as it promotes the spread of beneficial mutations that confer adaptation to intermediate stress levels [17,18].

The fitness landscape faced by the declining population is another critical factor in evolutionary rescue. While foundational theory was developed for quantitative traits affected by many loci [1], stochastic approaches focused on a single rescue allele with a fixed fitness effect [6,13]. Extending the latter approach to include a range of possible alleles of different effect sizes [10] elegantly coupled evolutionary rescue to Fisher’s geometric model (FGM) [19,20], a well-studied phenotype-to-fitness landscape [21–25]. This coupling naturally scales both the availability and effect size of rescue mutations with the degree of environmental stress faced by the population [10,26].

As a landscape on a continuous trait space, FGM assumes that all possible phenotypes are accessible by mutation. In realistic settings, however, only a finite set of mutations may contribute to evolutionary rescue. For example, a handful of well-characterized mutations confer resistance to antibiotics, as seen in bacterial populations [27–29]. In essence, evolutionary rescue occurs at a genotypic level.

How will predictions about evolutionary rescue change if we take this underlying genotypic basis into account? To address this question, we follow [30], which introduced a genotypic realization of FGM. Mutations in FGM change only the phenotype, so assigning a genetic locus to each mutation in order to produce a genotypic version seems straightforward, as illustrated for example by [31]. Yet, despite the additivity and smoothness of the genotype–phenotype map, this genotypic model typically displays multiple fitness optima, exhibiting behaviours that are distinct from those of the phenotypic FGM [30]. We use this genotypic approach to address evolutionary rescue, deriving analytical expressions for the probability that a population survives an adverse environmental event. We find that the rescue probability, averaged over many samples of the genotypic FGM, is always reduced when the genotypic basis of evolutionary rescue is taken into account.

## 2. The model

### 2.1. Fisher’s geometric model

In Fisher’s geometric model, a phenotype is represented as a point in an *N*-dimensional phenotypic space, where each axis corresponds to a specific trait [19,20]. Each trait is assumed to have an optimal value [32], and thus a single point in trait space defines the best possible phenotype. An individual’s fitness, *W*, is then a decreasing function of the distance between its phenotype and the optimum phenotype. Mathematically, this can be expressed as

2.1 $$W=W_{\max}\exp\left(-\frac{\sum_{i=1}^{N}{\left(q_{i}-O_{i}\right)}^{2}}{2\rho^{2}}\right).$$

**Figure 1.**
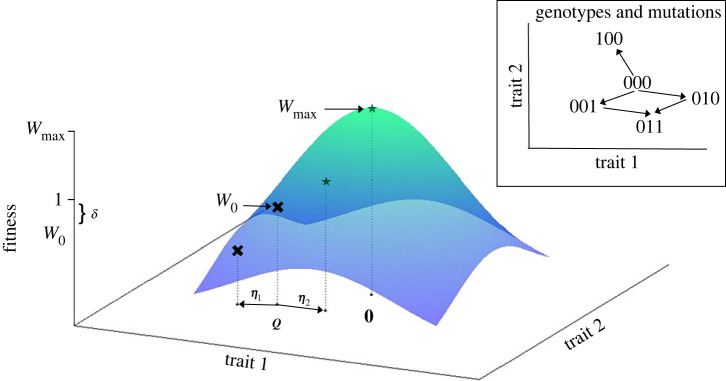
Fisher’s geometric model defines fitness (coloured surface) as a function of trait values, illustrated here for *N* = 2. The peak fitness *W*_max_ occurs at the optimal trait values, shown here as **0**. In an evolutionary rescue scenario, the wild-type phenotype, ***Q***, has fitness *W*_0_ < 1. Mutations occur as displacement vectors, ***η***. In this example, if mutation ***η***_1_ occurs, fitness is reduced. However if ***η***_2_ occurs, trait values move closer to the optimum and fitness is greater than unity (stars and black crosses indicate fitness values >1 and <1 respectively.) In the classic version of FGM, mutations can move the phenotype ***Q*** to any point in trait space. In contrast in the genotypic realization, the phenotype ***Q*** can only ‘jump’ by additive combinations of a finite set of ***η***_*i*_. An illustration for two traits, *N* = 2, and sequence size *L* = 3 is provided in the inset. Here the phenotype associated with genotype 011 is obtained from 000 by additively combining ***η***_2_ and ***η***_3_.

Here, ***Q*** = [*q*_1_, *q*_2_, …, *q*_*N*_] denotes the individual’s phenotype, while $O=[O_{1},O_{2},\ldots ,O_{N}]$ represents the optimum phenotype. The maximum possible fitness is denoted by *W*_max_, while *ρ* defines the width of the fitness function. See figure 1 for an illustration of the fitness surface.

Mutations can affect all trait values (universal pleiotropy); in particular, a single mutation is represented by a vector of phenotypic changes: $\varepsilon =[\varepsilon_{1},\;\varepsilon_{2},\ldots ,\varepsilon_{N}]$. The model further assumes that the effects of a mutation on the trait values are additive, i.e. $Q^{\prime }=Q+\varepsilon$, where ***Q′*** is the mutant phenotype. Here, we also make the standard assumption that the components $\varepsilon_{i}$ are normally distributed random variables with null mean and variance *σ*^2^ [33,34]. Thus, both selection and mutation are symmetrical, without correlations among traits (but see [35]).

In reality, all possible phenotypes may not be accessible via mutation. Following [30], we thus relax this assumption and consider a genotypic realization of FGM. In particular, we assume that a finite set of non-lethal mutations are available by mutation from the wild-type. For the evolutionary rescue scenarios considered here, we can think of this as the set of mutations that affect specific loci that are critical to rescue. For example, if a particular efflux pump must be upregulated for rescue, this could be the set of mutations that either up- or down-regulate efflux, without causing lethality. Similarly, if a receptor must escape binding to a new pathogen, we would consider the set of mutations that change the receptor binding properties, either increasing or decreasing affinity with the pathogen, without losing receptor function. Due to the assumption of universal pleiotropy, these mutations will affect other traits as well as the traits important for rescue.

We let *L* represent the number of available mutations in this set. The genetic state of each individual is thus simply represented as a binary sequence of size *L*, representing which of *L* possible mutations the individual carries (figure 1, inset). After choosing a wild-type phenotype (a point in trait space, ***Q***), we define the genetic state of the wild-type as *S* = (0, 0, …, 0) (since it carries no mutations). We then specify *L* available displacement (mutation) vectors ***η***_*k*_. Each element of each displacement vector ***η***_*k*_ is drawn from a normal distribution with mean zero and variance *σ*^2^ as in the classic FGM. The genetic state of each individual then reflects the absence or presence of each mutation, such that the phenotype of any of the 2^*L*^ possible ‘genotypes’ is obtained by simply adding any mutations carried by that type, ***η***_*k*_, to the wild-type phenotype ***Q***. As a result, only a finite set of points in trait space, and thus a finite set of fitness values, are accessible via the mutation vectors ***η***_*k*_, as illustrated in figure 1. Note that ε denotes a randomly drawn mutation vector as defined for the phenotypic FGM, while ***η***_*k*_ corresponds to one of the set of *L* mutation vectors in the genotypic model. Although identically distributed, these vectors are, crucially, drawn at different points in the stochastic process.

Finally, we note that the phenotypic effect of mutation *k* is constant and independent of the other mutations carried by the individual. There is thus no epistasis in trait space. However, the inherent nonlinearities of the phenotype–fitness map causes the emergence of epistasis at the level of fitness [36]. In fact, epistasis is a major determinant of the topography of these fitness landscapes, and consequently imposes constraints on the evolutionary process [31]. One important consequence of providing a discrete genotypic basis for the FGM is that the projection of the discrete genotypic space onto the continuous phenotype space can give rise to multiple distinct fitness maxima (see [30] for a detailed analysis).

### 2.2. Modelling evolutionary rescue

To model the rescue process analytically, we make several simplifying assumptions. In particular, we assume that the population initially consists of *N*_0_ genetically identical (wild-type) individuals. In this contribution, we therefore neglect the role of standing genetic variation (but see [13,16]) and predict only the contribution of de novo mutation to rescue. Similarly, we will assume that rescue occurs via a single mutation from a wild-type individual, neglecting the role of multiple mutations [26,37]; the latter assumption will be relaxed in the simulations to follow.

Starting with the phenotypic model, we assume that the wild-type phenotype ***Q*** is at distance $d=\sqrt{\sum q_{i}^{2}}$ from the fitness optimum; we assume (without loss of generality) that this optimum is at the origin. The wild-type absolute fitness is therefore

2.2 $$W_{0}=W_{\max}\exp\left(\frac{-d^{2}}{2\rho^{2}}\right).$$

To model a rescue process, we imagine that the environment has instantaneously changed, such that the wild-type population size is declining. We thus choose *d* sufficiently large such that the wild-type fitness, *W*_0_, is less than unity. We describe the ‘fitness drop’, *δ*, as the degree to which the wild-type is maladapted, *δ* = 1 − *W*_0_. We then assume that individuals reproduce asexually in discrete generations, producing a Poisson-distributed number of offspring with mean given by their absolute fitness. Mutations can occur during each birth event, where the mutation rate, per individual per replication, is given by *U*. As described above, we assume that *U* is sufficiently small that single mutations from the wild-type will dominate the rescue process.

As noted above, the key difference between the classic and genotypic versions of FGM is that in the genotypic model, only a finite set of possible mutations is accessible. These mutations are a random sample from the infinite set of mutations assumed in the classic FGM. In the analysis and simulations to follow, we present the expected behaviour over many such samples, that is, over many different sets of mutations. Since these mutations are sampled from the same mutational distribution as in the classic FGM, over many draws the distributions of mutations in the classic and genotypic FGM are identical. We also point out that in both models, the same number of mutations will occur during an evolutionary rescue scenario. It is thus not clear from the outset whether imposing a genotypic basis on FGM will have any effect on evolutionary rescue, and the results to follow were unexpected.

### 2.3. Simulating evolutionary rescue

To simulate this process, we begin with *N*_0_ individuals that carry no mutations, and thus all have the wild-type phenotype ***Q***, which we define as a point on the hypersurface of radius *d* from the origin. As described above, we choose *d* such that the wild-type fitness *W*_0_ < 1. Individuals are subject to selection and mutation. Thus, in each generation, each individual generates a Poisson-distributed number of offspring with mean *W*, where *W* refers to the absolute fitness determined by the individual’s phenotype (equation (2.1)). As described above, we consider non-overlapping generations.

Each offspring mutates with probability *U*. To simulate the classic (phenotypic) FGM, each time a mutation occurs we draw a new mutation vector, ε, such that the mutant phenotype is Q+ε. In the case of FGM with a genetic basis, we draw the *L* possible mutation vectors before the simulation begins. Each time a mutation occurs we then choose a random locus that will mutate; the locus in question will change from 0 in the parent to 1 in the offspring, or vice versa (back mutation is included in the simulations). A mutation at locus *k* will thus add (or subtract) the displacement vector ***η***_*k*_ to determine the offspring’s phenotype.

For each independent run, the simulation is stopped if either the population goes extinct, or if the number of individuals with fitness *W* > 1 surpasses 100 (our results are insensitive to this threshold as long as it is sufficiently large, as in [26]). The latter case is counted as an instance of evolutionary rescue. When rescue occurs in the genotypic model, we also record the mean number of mutations that are carried by individuals with *W* > 1. We also note that distances in this model can be rescaled by any one of the three length scales, *ρ*, *d* and *σ*. In the simulation results to follow, we take *ρ* = 1 and thus express distances in terms of the width of the fitness distribution around the optimum.

Unless stated otherwise, for each simulation result we drew 100 000 independent sets of mutations, and simulated a single rescue scenario for each mutational set. As compared with simulating replicate rescue scenarios over a smaller number of mutational sets, this procedure reduces statistical fluctuations. We also use the replicate approach, however, to examine the distribution of the rescue probabilities obtained across distinct sets of mutations.

## 3. Analytical predictions

### 3.1. Distribution of fitness effects of mutations

The distribution of fitness effects of single mutations (the DFE), where mutations are drawn randomly as described above, has a known form for this version of FGM [24,38]. The distribution of the relative growth rate, *m*/*m*_0_, has also been previously derived (appendix II.2 in [10]), where *m*_0_ is the Malthusian growth rate of the wild-type,

3.1 $$m_{0}=\log(W_{0})=\log(W_{\max})-\frac{d^{2}}{2\rho^{2}}.$$

In brief, we consider the growth rate of a lineage that carries one mutation, with displacement vector $\varepsilon =[\varepsilon_{1},\;\varepsilon_{2},\;\ldots ,\;\varepsilon_{N}]$. The phenotype of the mutant is Q+ε and the growth rate of the mutant is

3.2 $$\begin{array}{rl} m & =\log\left(W_{\max}\;e^{\left(-\sum {\left(q_{i}+\varepsilon_{i}\right)}^{2}\right)/2\rho^{2}}\right)\\ & =\log(W_{\max})-\frac{1}{2\rho^{2}}\sum {\left(q_{i}+\varepsilon_{i}\right)}^{2}\\ & =\log(W_{\max})-\frac{1}{2\rho^{2}}Y_{N}. \end{array}$$

Here, the random variable $Y_{N}=\sum {\left(q_{i}+\varepsilon_{i}\right)}^{2}$ is the sum of the squared values of *N* independent, normally distributed random variables, each with a different mean value (the *q*_*i*_). *Y*_*N*_ therefore follows a non-central *χ*-squared distribution, with *N* degrees of freedom and with non-centrality parameter given by $\sum q_{i}^{2}=d^{2}$ [39].

The probability density function for *Y*_*N*_ can therefore be written as

3.3 $$P(y)=\sum_{i=1}^{\infty }\frac{e^{-d^{2}/2}{\left(d^{2}/2\right)}^{i}}{i!}P_{N+2i}(y),$$

where $P_{j}(y)$ is the probability density function of a (standard) *χ*^2^ distribution with *j* degrees of freedom.

Using equation (3.2), we can also trivially rewrite equation (3.3) to give the probability density for the random variable *M*, the growth rate of a random mutant. For readability in the sections to follow, we will use *p*(*m*) to denote this probability density.

Finally, since the non-central *χ*^2^ distribution may be unfamiliar to the general scientific reader, we present a more accessible derivation of *p*(*m*) in the electronic supplementary material.

### 3.2. Evolutionary rescue: phenotypic Fisher's geometric model

To estimate the rescue probability in the phenotypic model, we follow the standard approach [6,13], first estimating the overall number of de novo mutations that occur while the wild-type population is en route to extinction. Since the wild-type population decays as a geometric series, this is given by

3.4 $$A=UN_{0}\sum_{i=1}^{\infty }W_{0}^{i}=\frac{UN_{0}W_{0}}{1-W_{0}},$$

where the the index of summation begins at one because our model assumes discrete generations in which mutation follows birth.

We define a rescue mutation as a displacement vector that, when added to the wild-type phenotype, results in a positive growth rate (*m* > 0). The probability that a randomly chosen mutation is a rescue mutation, and survives extinction when rare, is then given by

3.5 $$\tilde{\pi }=\int_{m=0}^{\hat{m}}p(m)\pi (m)\;dm,$$

where $\hat{m}=\log(W_{\max})$ is the maximum possible growth rate, and *π*(*m*) is the probability that a mutant lineage with growth rate *m* survives extinction. Although several good approximations to *π*(*m*) are available [40], in our model growth is density-independent, and thus the growth of the mutant subpopulation is a branching process. In the results to follow we can therefore use the exact result, computing *π*(*m*) numerically as the fixed point of the probability generating function for Poisson-distributed offspring: *π*(*m*) = 1 − exp (−e^*m*^*π*(*m*)) ([41], pp. 145–150).

The probability of evolutionary rescue in the phenotypic model, due to de novo mutation, is then approximated by [6,13]

3.6 $$P_{\mathrm{rescue}}=1-{\left(1-\tilde{\pi }\right)}^{A}$$

3.7 $$\;\approx 1-e^{-A\tilde{\pi }}.$$

Importantly, the compact form of equation (3.6) is possible because each of the *A* mutations constitutes a new random draw from the distribution of growth rates, *p*(*m*). This is the main feature that does not hold in the genotypic model.

### 3.3. Evolutionary rescue: genotypic Fisher's geometric model

In the genotypic model, the number of available displacement vectors (mutations) is limited. Since the expected number of de novo mutations that occur before extinction is *A* and all mutations are equally likely, the number of de novo occurrences of each displacement vector is Poisson distributed with expected value *A*/*L*.

Considering the distribution of growth rates of single mutations, *p*(*m*), we define the rescue fraction, *f*, as the probability that a displacement vector is a potential rescue mutation,

3.8 $$f=\int_{m=0}^{\hat{m}}p(m)\;dm.$$

For displacement vectors that are potential rescue mutations, we also know that their growth rates are distributed with probability density

$$\;p(m|m>0)=\frac{\;p(m)}{\int_{0}^{\hat{m}}p(m)\;dm}=\frac{\;p(m)}{f}\;\;\text{for}\;m\in (0,\hat{m}).$$

We first consider a single displacement vector that is a potential rescue mutation. The probability that it fails to rescue the population is given by the probability that it occurs de novo *i* times, and all *i* independent lineages go extinct: (1 − *π*(*m*))^*i*^. We can then condition on the probability that this mutation occurs *i* times, sum over all *i* and integrate over all possible growth rates for a rescue mutation. The complement of this integral yields *R*, the probability that a single displacement vector rescues the population,

3.9 $$R=1-\frac{1}{f}\int_{m=0}^{\hat{m}}p(m)\sum_{i=0}^{\infty }\frac{e^{-\lambda }\lambda^{i}}{i!}{\left(1-\pi (m)\right)}^{i}\;dm,$$

where *λ* = *A*/*L*. Here we see the difference between the phenotypic and genotypic rescue scenarios, in that a mutation with a particular growth advantage can reoccur *i* times in the genotypic model. Thus, rather than considering the probability that a single de novo mutation survives and rescues the population, we compute the probability that a single potential rescue mutation actually rescues the population. Conveniently, the infinite sum in *R* simplifies to yield:

3.10 $$R=1-\frac{1}{f}\int_{m=0}^{\hat{m}}p(m)\exp\left(\frac{-\pi (m)A}{L}\right)dm.$$

Since there are *L* displacement vectors in total, the probability that *k* of the *L* displacement vectors are potential rescue mutations is given by the binomial distribution with parameters *L* and *f*. To estimate the probability that at least one of these vectors rescues the population, we sum over *k*, the number of potential rescue mutations,

3.11 $$P_{\mathrm{rescue},L}=1-\sum_{k=0}^{L}\begin{array}{c} L\\ k \end{array}f^{k}{\left(1-f\right)}^{L-k}{\left(1-R\right)}^{k},$$

and use the binomial theorem to simplify the above equation to

3.12 $$P_{\mathrm{rescue},L}=1-{\left(1-Rf\right)}^{L}$$

3.13 $$\;\approx 1-e^{-RfL}.$$

Equation (3.12) has a natural intuitive interpretation. If we consider each of the *L* displacement vectors, *f* gives the probability that each of these is a potential rescue mutation. The product *Rf* gives the probability that this vector does indeed rescue the population. Therefore, 1 − *Rf* is the probability that one of the *L* displacement vectors fails to rescue the population, and the expression for *P*_rescue_,*_L_* follows.

Finally, combining equations (3.10) and (3.12) we have

3.14 $$\begin{array}{r} P_{\mathrm{rescue},L}=1-{\left(1-f+\;\int_{m=0}^{\hat{m}}p(m)\exp\left(\frac{-\pi (m)A}{L}\right)dm\right)}^{L}. \end{array}$$

Defining *X*_*L*_ as the complement $1-P_{\mathrm{rescue},L}$, it is straightforward to show that

3.15 $$\lim_{L\to \infty }\log X_{L}=-A\int_{m=0}^{\hat{m}}p(m)\pi (m)\;dm,$$

and thus we find that

3.16 $$\lim_{L\to \infty }X_{L}=\exp\left(-A\int_{0}^{\hat{m}}p(m)\pi (m)\;dm\right)=\exp\left(-A\int_{-\infty }^{\hat{m}}p(m)\pi (m)\;dm\right),$$

under the assumption that the fixation of deleterious mutations during evolutionary rescue is negligible, i.e. *π*(*m*) = 0 for *m* < 0. Under this assumption, in the limit as *L* → ∞, equation (3.12) yields equation (3.7). In other words, the probability of evolutionary rescue in the genotypic model approaches the rescue probability in the phenotypic model as the number of available mutations approaches infinity.

## 4. Results

In the results to follow, we evaluate the distribution of growth rates, *p*(*m*), numerically and then use either equation (3.6) or equation (3.12) to predict rescue probabilities in the phenotypic or genotypic realizations of FGM, respectively. We confirm all analytical results with simulations as described above.

**Figure 2.**
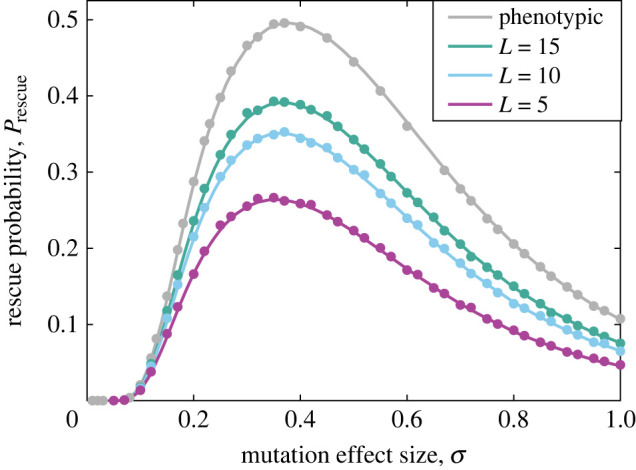
Rescue probability versus mutation effect size. The probability of evolutionary rescue for the classic, phenotypic FGM (grey lines, equation (3.6)) and for the genotypic model (equation (3.12)) is plotted versus the mutation effect size, *σ*. We find that the rescue probability is reduced in the genotypic model, decreasing with the number of available mutations, *L* (L=15, 10 and 5 plotted in green, blue and purple, respectively). Filled circles are simulation results (error bars are similar to or smaller than symbol heights and have been omitted). Other parameters are: *N* = 5, *U* = 10^−3^, *δ* = 0.2, *ρ* = 1.

Figure 2 shows the probability of rescue as a function of mutation effect size, *σ*, where the distance between the wild-type population and the fitness optimum is fixed. This figure illustrates several trends that will be repeated throughout our results. First, we find that when a finite number of mutations are available (genotypic model), rescue is less likely than in the classic, phenotypic FGM. Second, we see that rescue becomes less likely as the number of available mutations, *L*, is reduced. Third, we see that incorporating genotypic constraints in FGM does not change the overall behaviour of evolutionary rescue as a function of other parameters. Here for example both *P*_rescue_ and $P_{\mathrm{rescue},L}$ are unimodal functions of *σ*, showing that for a fixed distance from the optimum, a single ‘best’ mutation size is most likely to achieve evolutionary rescue, whether or not genotypes are included in the model. Finally, we note that our analytical approach assumes that only single mutations from the wild-type contribute to rescue. Although this assumption is relaxed in the simulations, the agreement between analytical predictions (lines) and simulation results (filled circles) is striking. We note that at the parameter values illustrated here, in particular at this mutation rate, the average number of mutations in individuals with positive growth rates at the end of a simulation is approximately 1.1, so this assumption is not often violated (see figure 5, inset, for further discussion on this point).

**Figure 3.**
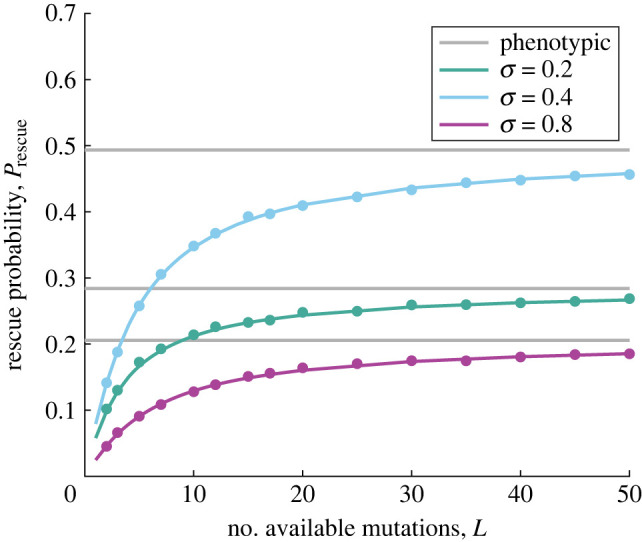
Rescue probability versus the number of available mutations. The probability of evolutionary rescue for the classic, phenotypic FGM (grey lines, equation (3.6)) and for the genotypic model (equation (3.12)) is plotted versus the number of available mutations, *L*. We find that the rescue probability is reduced in the genotypic model, but approaches the prediction for the phenotypic model as *L* grows large. Filled circles are simulation results (error bars are similar to or smaller than symbol heights and have been omitted). Results are shown for different values of mutation size effect, *σ*, as indicated in the legend. Other parameters are: *N* = 5, *U* = 10^−3^, *δ* = 0.2, *ρ* = 1.

In figure 3, we further illustrate the influence of the number of available mutations on $P_{\mathrm{rescue},L}$. Results are presented for small, intermediate and large mutation effect sizes. As predicted, including genotypic constraints always reduces rescue probability, and $P_{\mathrm{rescue},L}$ grows monotonically with *L*. More importantly, we confirm that as *L* → ∞, the rescue probability in the genotypic model approaches the prediction under Fisher’s classic geometric model (grey horizontal lines).

**Figure 4.**
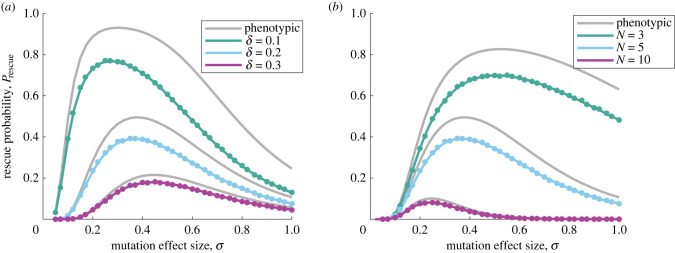
Rescue probability versus mutation effect size as the distance to the fitness optimum or the dimensionality of trait space varies. The probability of evolutionary rescue for the classic, phenotypic FGM (grey lines, equation (3.6)) and for the genotypic model (equation (3.12)) is plotted versus the mutation effect size, *σ*. Again the rescue probability is reduced in the genotypic model, but retains the overall behaviour of the phenotypic model. In (*b*), note that the magnitude of each mutation vector is not rescaled as *N* varies; the average magnitude thus increases with *N* for a fixed *σ*. This explains the left shift in the optimal value of *σ* for higher *N*. Filled circles are simulation results (error bars are similar to or smaller than symbol heights and have been omitted). Other parameters are: *N* = 5 (*a*), *δ* = 0.2 (*b*), *U* = 10^−3^, *ρ* = 1 and *L* = 15.

As discussed previously, an essential factor in the likelihood of evolutionary rescue is the initial level of stress the population experiences [1,10,11]. In our model, this factor is reflected in the parameter *δ*, which describes the degree to which the wild-type is maladapted after the environmental change. Figure 4*a* shows, as expected, that the rescue probability drops considerably as *δ* increases. However, we also notice that when *δ* is large, the effect of a finite genome on *P*_rescue_ is weakened, both in absolute terms and relatively. Figure 4*b* shows the effect of the number of traits (dimensionality of trait space) on *P*_rescue_. When the number of independent traits contributing to fitness increases, the chance of rescue is reduced; again the genotypic model does not change the overall pattern but reduces rescue probabilities at any parameter values.

**Figure 5.**
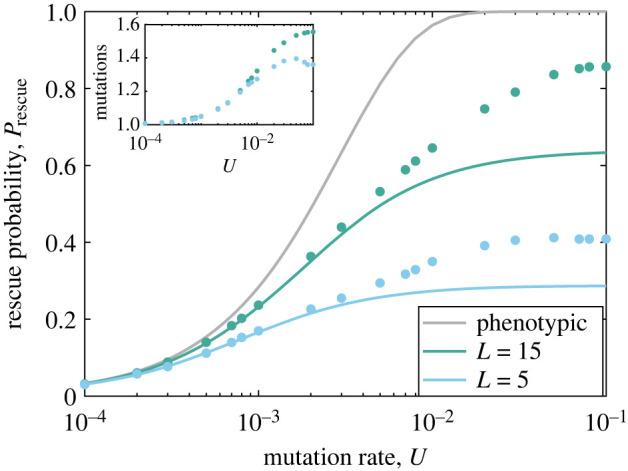
Rescue probability versus mutation rate. The probability of evolutionary rescue for the classic, phenotypic FGM (grey line, equation (3.6)) and for the genotypic model at *L* = 5 and *L* = 15 (equation (3.12)) is plotted versus the mutation rate, *U*. Filled circles are simulation results (error bars are similar to or smaller than symbol heights and have been omitted). The inset shows the number of mutations present, on average, in individuals with positive growth rates at the end of the simulation. As *U* increases, multiple mutations contribute to the rescue process, and the analytical approximation fails. However, again we see that rescue is reduced in the genotypic model, and increases with *L*. Other parameters are: *σ* = 0.2, *N* = 5, *δ* = 0.2 and *ρ* = 1.

Figure 5 shows rescue probability as a function of mutation rate. As the influx of de novo mutations is proportional to the mutation rate *U*, the monotonic growth of rescue probabilities with *U* is not surprising. Previous work on the classic FGM has already proven that a sufficiently high mutation rate can ensure the persistence of the population [10,42]; we illustrate this effect in figure 5, where *P*_rescue_ approaches unity as *U* exceeds approximately 0.01. Coloured lines show our analytical predictions for $P_{\mathrm{rescue},L}$, which substantially underestimate simulated rescue probabilities at high mutation rates. This occurs because the assumption that only single mutational steps from the wild-type contribute to rescue is no longer valid at high mutation rates. The inset of figure 5 plots the number of mutation vectors present, on average, in individuals with positive growth rates at the end of each simulation run, demonstrating how this assumption is violated as *U* grows.

An interesting feature is suggested by these results as well: the assumption of a finite number of available mutations imposes an upper bound on rescue probabilities, such that even at high mutation rates, the persistence of the population is no longer guaranteed. The smaller the set of available mutations, the lower the upper bound on the rescue probability. This makes intuitive sense because even at high mutation rates, there is at least some chance that no combination of the *L* displacement vectors produces a genotype with a positive growth rate. Since an approach that includes multiple mutations is beyond the scope of this work, we leave this interesting observation for future work.

**Figure 6.**
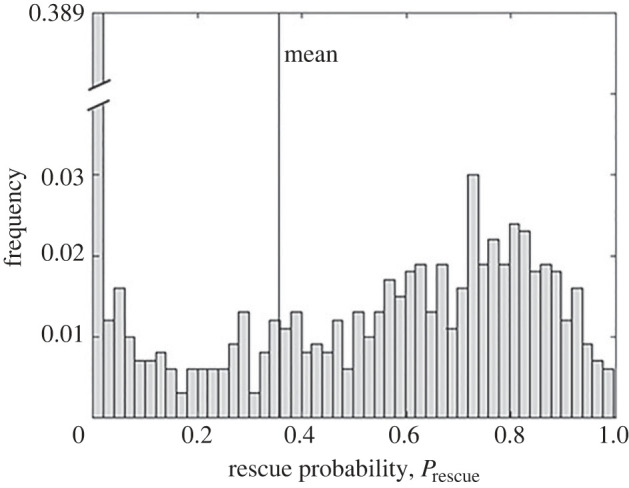
Distribution of rescue probabilities across 1000 distinct draws of the mutational vectors, with the rescue scenario simulated 1000 times per set. The mean rescue probability was 0.355 (vertical line) with standard deviation 0.349. Note that the *y*-scale is broken for clarity. The parameters values are: *σ* = 0.2, *L* = 10, *N* = 5, *δ* = 0.2 and *ρ* = 1.

In figures 2–5, the probability of rescue was obtained by averaging over 100 000 independent sets of mutations, where a single rescue scenario was simulated using each set. In figure 6, we show the dispersion of rescue probabilities over an ensemble of 1000 sets of mutations, where for each set, we simulated 1000 independent evolutionary rescue scenarios. Here we see a broad spectrum of probabilities, covering the whole range of possible values. A key observation here is that a substantial fraction of mutational draws do not allow, or only rarely allow rescue (broken bar on left); this fraction may be a key driver of rescue probability when considering a finite number of loci.

## 5. Discussion

Evolutionary rescue (ER) has been the subject of intense study in the evolutionary biology community [2,3]. The seminal work of Golmukiewicz and Holt introduced ER via many mutations of small effect [1], while Orr and Unckless quantified the probability of evolutionary rescue via a single mutation [6,13]. In the latter approach, the wild-type allele is assumed to have absolute fitness 1 − *δ* in the new environment. The population’s survival relies on a beneficial allele—either arising by de novo mutation or existing in standing genetic variation—that confers an absolute fitness 1 − *δ* + *s*, where *s* > *δ*.

As mentioned in the Introduction, the level of environmental stress, reflected in the parameter *δ*, has both direct and indirect effects on evolutionary rescue. Beyond influencing the demographic characteristics of a population, a stressful environment can also impact the rate of adaptation [43]. This occurs because the degree of maladaptation of the wild-type can influence factors such as the rate and distribution of mutation effects on traits [35]. These indirect effects highlight the importance of considering the interplay between environmental stress and the rate of adaptation in ER studies. Following [10], here we use Fisher’s geometric model to study a full distribution of fitness effects of mutations (DFE), and to provide a framework in which the DFE changes in a natural way for populations experiencing different levels of stress.

Previous work introduced a genotypic realization of FGM that allows for a finite set of beneficial mutations [30]. These authors demonstrate that the discrete nature of genotypic space gives rise to intriguing features in the emergent fitness landscape. In particular, although the phenotypic landscape is single-peaked and smooth, the fitness landscape in the genotypic realization exhibits multiple fitness peaks, and the number of peaks increases exponentially with the size of the genome [30].

In this contribution, we use the genotypic realization of FGM to study evolutionary rescue via de novo mutation. Importantly, during rescue under the phenotypic FGM, each mutation comprises a random draw from the DFE. This assumption no longer holds for the genotypic model, as now, the number of available mutations is constrained to a finite set of size *L*. We derive the probability that at least one of these *L* mutation vectors rescues the population. Our analytical predictions have a clear intuitive interpretation and are validated by simulation. Our theory breaks down when the assumption that only single mutations contribute to rescue is broken, i.e. in the limit of high mutation rates.

Our results demonstrate that the probability of evolutionary rescue is always reduced when the genetic basis of the rescue process is taken into account. This finding is somewhat counterintuitive, given that the rate at which mutations occur is unchanged, that all mutations are drawn from the same distribution, and that genotypic results show the average over many such draws. Whenever a finite set of mutations is drawn, however, there is a non-zero probability that none of the mutations improves fitness. Our results show that with all other parameters held constant, the rescue probability can vary widely for different sets of mutations (figure 6), and can include a large fraction of mutational sets for which rescue is impossible or unlikely. In addition, we note that the largest value of a set of *L* draws from any distribution increases with *L* [44]. Thus for parameter regimes in which rescue is driven by rare beneficial mutations, we might expect that allowing only *L* draws from the mutation vector distribution will reduce rescue probabilities.

Consistent with this line of reasoning, the rescue probability increases when the set of available mutations grows larger. In fact, we demonstrate analytically that the rescue probability in the classic FGM is recovered in the limit as *L* → ∞. This is interesting since the topographical properties of this fitness landscape, such as the number of local maxima, do not approach those of the classic FGM in this limit [30].

Since *L*, the number of available mutations, may be large (see [45] for discussion), it is important to discuss the circumstances under which the genotypic basis of ER might be relevant to quantitative predictions. In other words, when will *P*_rescue_ and $P_{\mathrm{rescue},L}$ differ substantially? High degrees of parallel evolution have been reported in microbial evolution experiments [46–50], as reviewed by Bailey *et al*. [51] and Bolnick *et al*. [52]. While parallelism is highest at the level of phenotype, substantial parallel evolution has been observed at the level of individual genes [48,49], even across bacterial phyla and clinical isolates [50]. High degrees of parallelism in individual mutations have also been observed in the adaptation of both bacteriophages [46] and mitochondrial DNA in malaria [47]. Thus at least for microbial populations, a relatively small number of mutations may contribute to evolutionary rescue. The effect of a finite set of available mutations is likely to be most important in small, highly constrained genomes such as viruses. For such pathogens, our work demonstrates that classic ER predictions based on the phenotypic FGM will overestimate rescue probabilities; classic predictions will thus provide conservative estimates of treatment doses that are predicted to eliminate an infection.

We also note that *L* is defined as the total number of available, non-lethal mutations that affect the traits of interest. Of these, fraction *f* are potential rescue mutations. This formalism allows us to use FGM directly to compute the DFE. Returning to the example of a receptor protein, we might imagine *L* nucleotide substitutions that affect binding properties. With some combination of those nucleotides, the receptor is best able to escape the pathogen; with another combination, the receptor is least able to escape. The wild-type individual in our model does not begin at the nadir of this fitness landscape; both beneficial and deleterious mutations are available. As with rescue mutations, the number of available deleterious mutations scales directly in our model with the total number of available mutations, *L*.

It is also possible, however, that while the number of potential rescue mutations remains small, the number of available deleterious mutations could be extremely large and may not scale with *L*. Since this effect is captured in FGM only very close to the optimum, further model development would be required to study this situation.

The genetic basis of ER has previously been studied within the framework of quantitative genetics [53]. In that study, ER was found to be less likely when more loci contributed to fitness. While this seems at odds with our overriding conclusion that ER increases with *L*, such comparisons depend critically on what is being held constant. In [53], the initial population fitness and maximum growth rate are held constant, such that the selective effect per mutation falls as the number of loci increases. By contrast, we compare mutations with the same average effect size, but vary the number of such mutations that are available. This comparison points to rich open questions at the interface of quantitative- and population-genetic models of ER.

As outlined above, we find that rescue probabilities are reduced when their genotypic basis is taken into account. Nonetheless, the overall qualitative behaviour of ER as a function of other model parameters remains unchanged. For example, we show that the rescue probability is a single-peaked function of the mutation effect size *σ*. This phenomenon has been previously observed (e.g. [54,55]) and can be understood as follows. The probability that a mutation is beneficial approaches 1/2 when the effect size is very small, as compared with the distance to the optimum [33,56]. Nonetheless, mutations with a small effect are unlikely to promote rescue if the initial maladaptation level of the population is high. Mutations that strongly affect traits, in contrast, are rare and are also less likely to be beneficial. The balance of these effects implies that mutations of intermediate effect size are the most likely to contribute to population rescue. Our results suggest that the mutation effect size that maximizes *P*_rescue_ is invariant with the number of available mutations, *L*, but strongly influenced by the stress level *δ*. Indeed, levels of extinction risk and population growth rates are known to change the spectrum of effect size for established mutations in the classic FGM [54,55]. In particular, fewer and larger effect mutations are more likely to fix under high extinction risk, an effect that is even more pronounced when the number of available loci is small [55].

Another intriguing prediction of the genotypic model is the observation of an upper bound on the rescue probability. While rescue is essentially assured in the classic FGM at high mutation rates, when only a finite set of mutations is available, the rescue probability plateaus below unity as the mutation rate increases. The existence of an upper bound for the rescue probability follows from the fact that there exists a non-zero probability that none of the *L* mutation vectors restores a positive growth rate.

To allow for analytical progress, we studied ER via de novo mutation only. Standing genetic variation is another important contributor to evolutionary rescue [13,16] that can produce counterintuitive effects in the rescue process [9]. We also narrowed our focus to the effects of single mutations from the wild-type (but see [26]). Since evolutionary rescue is, in essence, a genotypic phenomenon, relaxing these assumptions while retaining a genotypic fitness landscape is a clear avenue for future work.

## Data Availability

Data is available from the Dryad Digital Repository: https://datadryad.org/stash/share/Y21kHew-FrMuXhss2NaWnLjjgD5YIzHk9oiMObpWAAg [57]. Supplementary material is available online [58].

## References

[1] Gomulkiewicz R, Holt RD. 1995 When does evolution by natural selection prevent extinction? Evolution **49**, 201-207. (10.2307/2410305) 28593677

[2] Alexander HK, Martin G, Martin OY, Bonhoeffer S. 2014 Evolutionary rescue: linking theory for conservation and medicine. Evol. Appl. **7**, 1161-1179. (10.1111/eva.12221) 25558278 PMC4275089

[3] Bell G. 2017 Evolutionary rescue. Ann. Rev. Ecol. Evol. Syst. **48**, 605-627. (10.1146/annurev-ecolsys-110316-023011)

[4] Uecker H, Hermisson J. 2011 On the fixation process of a beneficial mutation in a variable environment. Genetics **188**, 915-930. (10.1534/genetics.110.124297) 21652524 PMC3176092

[5] Bell G, Gonzalez A. 2009 Evolutionary rescue can prevent extinction following environmental change. Ecol. Lett. **12**, 942-948. (10.1111/j.1461-0248.2009.01350.x) 19659574

[6] Orr HA, Unckless RL. 2008 Population extinction and the genetics of adaptation. Am. Nat. **172**, 160-169. (10.1086/589460) 18662122

[7] Flather CH, Hayward GD, Beissinger SR, Stephens PA. 2011 Minimum viable populations: is there a ‘magic number’ for conservation practitioners?. Trends Ecol. Evol. **26**, 307-316. (10.1016/j.tree.2011.03.001) 21458878

[8] Bell G. 2013 Evolutionary rescue and the limits of adaptation. Phil. Trans. R. Soc. B **368**, 20120080. (10.1098/rstb.2012.0080) 23209162 PMC3538447

[9] Tanaka MM, Wahl LM. 2022 Surviving environmental change: when increasing population size can increase extinction risk. Proc. R. Soc. B **289**, 20220439. (10.1098/rspb.2022.0439) PMC9156903 35642362

[10] Anciaux Y, Chevin LM, Ronce O, Martin G. 2018 Evolutionary rescue over a fitness landscape. Genetics **209**, 265-279. (10.1534/genetics.118.300908) 29535150 PMC5937192

[11] Harmand N, Gallet R, Jabbour-Zahab R, Martin G, Lenormand T. 2017 Fisher’s geometrical model and the mutational patterns of antibiotic resistance across dose gradients. Evolution **71**, 23-37. (10.1111/evo.13111) 27805262

[12] Marrec L, Bitbol AF. 2020 Adapt or perish: evolutionary rescue in a gradually deteriorating environment. Genetics **216**, 573-583. (10.1534/genetics.120.303624) 32855198 PMC7536851

[13] Orr HA, Unckless RL. 2014 The population genetics of evolutionary rescue. PLoS Genet. **10**, e1004551. (10.1371/journal.pgen.1004551) 25121960 PMC4133041

[14] Wargo AR, Huijben S, De Roode JC, Shepherd J, Read AF. 2007 Competitive release and facilitation of drug-resistant parasites after therapeutic chemotherapy in a rodent malaria model. Proc. Natl Acad. Sci. USA **104**, 19 914-19 919. (10.1073/pnas.0707766104) PMC2148397 18056635

[15] Pena-Miller R, Laehnemann D, Jansen G, Fuentes-Hernandez A, Rosenstiel P, Schulenburg H, Beardmore R. 2013 When the most potent combination of antibiotics selects for the greatest bacterial load: the smile-frown transition. PLoS Biol. **11**, e1001540. (10.1371/journal.pbio.1001540) 23630452 PMC3635860

[16] Uecker H, Otto SP, Hermisson J. 2014 Evolutionary rescue in structured populations. Am. Nat. **183**, E17-E35. (10.1086/673914) 24334746

[17] Bell G, Gonzalez A. 2011 Adaptation and evolutionary rescue in metapopulations experiencing environmental deterioration. Science **332**, 1327-1330. (10.1126/science.1203105) 21659606

[18] Bourne EC, Bocedi G, Travis JM, Pakeman RJ, Brooker RW, Schiffers K. 2014 Between migration load and evolutionary rescue: dispersal, adaptation and the response of spatially structured populations to environmental change. Proc. R. Soc. B **281**, 20132795. (10.1098/rspb.2013.2795) PMC3906938 24452022

[19] Fisher RA. 1930 The genetical theory of natural selection. London, UK: Claredon Press.

[20] Orr HA. 2005 The genetic theory of adaptation: a brief history. Nat. Rev. Genet. **6**, 119-127. (10.1038/nrg1523) 15716908

[21] Bürger R, Lynch M. 1995 Evolution and extinction in a changing environment: a quantitative-genetic analysis. Evolution **49**, 151-163. (10.1111/j.1558-5646.1995.tb05967.x) 28593664

[22] Gordo I, Campos PR. 2013 Evolution of clonal populations approaching a fitness peak. Biol. Lett. **9**, 20120239. (10.1098/rsbl.2012.0239) 22764110 PMC3565474

[23] Matuszewski S, Hermisson J, Kopp M. 2014 Fisher’s geometric model with a moving optimum. Evolution **68**, 2571-2588. (10.1111/evo.12465) 24898080 PMC4285815

[24] Martin G. 2014 Fisher’s geometrical model emerges as a property of complex integrated phenotypic networks. Genetics **197**, 237-255. (10.1534/genetics.113.160325) 24583582 PMC4012483

[25] Martin G, Elena SF, Lenormand T. 2007 Distributions of epistasis in microbes fit predictions from a fitness landscape model. Nat. Genet. **39**, 555-560. (10.1038/ng1998) 17369829

[26] Osmond MM, Otto SP, Martin G. 2020 Genetic paths to evolutionary rescue and the distribution of fitness effects along them. Genetics **214**, 493-510. (10.1534/genetics.119.302890) 31822480 PMC7017017

[27] Weinreich DM, Delaney NF, DePristo MA, Hartl DL. 2006 Darwinian evolution can follow only very few mutational paths to fitter proteins. Science **312**, 111-114. (10.1126/science.1123539) 16601193

[28] Das SG, Direito SO, Waclaw B, Allen RJ, Krug J. 2020 Predictable properties of fitness landscapes induced by adaptational tradeoffs. Elife **9**, e55155. (10.7554/eLife.55155) 32423531 PMC7297540

[29] Marcusson LL, Frimodt-Møller N, Hughes D. 2009 Interplay in the selection of fluoroquinolone resistance and bacterial fitness. PLoS Pathog. **5**, e1000541. (10.1371/journal.ppat.1000541) 19662169 PMC2714960

[30] Hwang S, Park SC, Krug J. 2017 Genotypic complexity of Fisher’s geometric model. Genetics **206**, 1049-1079. (10.1534/genetics.116.199497) 28450460 PMC5499163

[31] Bank C. 2022 Epistasis and adaptation on fitness landscapes. Ann. Rev. Ecol. Evol. Syst. **53**, 457-479. (10.1146/annurev-ecolsys-102320-112153)

[32] Waxman D. 2006 Fisher’s geometrical model of evolutionary adaptation—beyond spherical geometry. J. Theor. Biol. **241**, 887-895. (10.1016/j.jtbi.2006.01.024) 16530790

[33] Tenaillon O. 2014 The utility of Fisher’s geometric model in evolutionary genetics. Annu. Rev. Ecol. Evol. Syst. **45**, 179-201. (10.1146/annurev-ecolsys-120213-091846) 26740803 PMC4699269

[34] Blanquart F, Achaz G, Bataillon T, Tenaillon O. 2014 Properties of selected mutations and genotypic landscapes under Fisher’s geometric model. Evolution **68**, 3537-3554. (10.1111/evo.12545) 25311558 PMC4326662

[35] Martin G, Lenormand T. 2006 A general multivariate extension of Fisher’s geometrical model and the distribution of mutation fitness effects across species. Evolution **60**, 893-907. (10.1111/j.0014-3820.2006.tb01169.x) 16817531

[36] Diaz-Colunga J, Skwara A, Gowda K, Diaz-Uriarte R, Tikhonov M, Bajic D, Sanchez A. 2023 Global epistasis on fitness landscapes. Phil. Trans. R. Soc. B **378**, 20220053. (10.1098/rstb.2022.0053) 37004717 PMC10067270

[37] Wilson BA, Pennings PS, Petrov DA. 2017 Soft selective sweeps in evolutionary rescue. Genetics **205**, 1573-1586. (10.1534/genetics.116.191478) 28213477 PMC5378114

[38] Martin G, Lenormand T. 2015 The fitness effect of mutations across environments: Fisher’s geometrical model with multiple optima. Evolution **69**, 1433-1447. (10.1111/evo.12671) 25908434

[39] Evans M, Hastings N, Peacock B. 2000 Statistical distributions, 3rd edn. New York, NY: John Wiley & Sons.

[40] Patwa Z, Wahl L. 2008 The fixation probability of beneficial mutations. J. R. Soc. Interface **5**, 1279-1289. (10.1098/rsif.2008.0248) 18664425 PMC2607448

[41] Allen LJS. 2010 An introduction to stochastic processes with applications to biology, 2nd edn. New York, NY: Chapman and Hall/CRC.

[42] Anciaux Y, Lambert A, Ronce O, Roques L, Martin G. 2019 Population persistence under high mutation rate: from evolutionary rescue to lethal mutagenesis. Evolution **73**, 1517-1532. (10.1111/evo.13771) 31134614

[43] Agrawal AF, Whitlock MC. 2010 Environmental duress and epistasis: how does stress affect the strength of selection on new mutations? Trends Ecol. Evol. **25**, 450-458. (10.1016/j.tree.2010.05.003) 20538366

[44] de Haan L, Ferreira A 2010 Extreme value theory: an introduction. Springer Series in Operations Research and Financial Engineering, 1st edn. Berlin, Germany: Springer.

[45] Das SG, Krug J. 2022 Unpredictable repeatability in molecular evolution. Proc. Natl Acad. Sci. USA **119**, e2209373119. (10.1073/pnas.2209373119) 36122210 PMC9522380

[46] Wichman HA, Badgett MR, Scott LA, Boulianne CM, Bull JJ. 1999 Different trajectories of parallel evolution during viral adaptation. Science **285**, 422-424. (10.1126/science.285.5426.422) 10411508

[47] Musset L, Le Bras J, Clain J. 2007 Parallel evolution of adaptive mutations in *Plasmodium falciparum* mitochondrial DNA during Atovaquone-Proguanil treatment. Mol. Biol. Evol. **24**, 1582-1585. (10.1093/molbev/msm087) 17488741

[48] Wong A, Kassen R. 2011 Parallel evolution and local differentiation in quinolone resistance in *Pseudomonas aeruginosa*. Microbiology **157**, 937-944. (10.1099/mic.0.046870-0) 21292748

[49] Tenaillon O et al. 2016 Tempo and mode of genome evolution in a 50 000-generation experiment. Nature **536**, 165-170. (10.1038/nature18959) 27479321 PMC4988878

[50] Scribner MR, Santos-Lopez A, Marshall CW, Deitrick C, Cooper VS. 2020 Parallel evolution of tobramycin resistance across species and environments. mBio **11**, e00932-20. (10.1128/mBio.00932-20) 32457248 PMC7251211

[51] Bailey SF, Blanquart F, Bataillon T, Kassen R. 2017 What drives parallel evolution? BioEssays **39**, 1-9. (10.1002/bies.201600176) 27859467

[52] Bolnick DI, Barrett RD, Oke KB, Rennison DJ, Stuart YE. 2018 (Non)parallel evolution. Annu. Rev. Ecol. Evol. Syst. **49**, 303-330. (10.1146/annurev-ecolsys-110617-062240)

[53] Gomulkiewicz R, Holt RD, Barfield M, Nuismer SL. 2010 Genetics, adaptation, and invasion in harsh environments. Evol. Appl. **3**, 97-108. (10.1111/j.1752-4571.2009.00117.x) 25567911 PMC3352474

[54] McDonough Y, Connallon T. 2023 Effects of population size change on the genetics of adaptation following an abrupt change in environment. Evolution **77**, qpad103. (10.1093/evolut/qpad103) 37279538

[55] Yamaguchi R, Wiley B, Otto SP. 2022 The phoenix hypothesis of speciation. Proc. R. Soc. B **289**, 20221186. (10.1098/rspb.2022.1186) PMC9667362 36382528

[56] Ram Y, Hadany L. 2015 The probability of improvement in Fisher’s geometric model: a probabilistic approach. Theor. Popul. Biol. **99**, 1-6. (10.1016/j.tpb.2014.10.004) 25453607

[57] Wahl LM, Campos RA. 2023 Data from: Evolutionary rescue on genotypic fitness landscapes. Dryad Digital Repository. (10.5061/dryad.cz8w9gj84) PMC10645506 37963553

[58] Wahl LM, Campos RA. 2023 Evolutionary rescue on genotypic fitness landscapes. Figshare. (10.6084/m9.figshare.c.6916135) PMC10645506 37963553

